# Loss of Pregnancy–Related Medicaid Coverage Is Associated With Unmet Need for Health Care, Medical Financial Hardship, and Lower Health Care Use in Postpartum Women

**DOI:** 10.1002/puh2.70044

**Published:** 2025-03-11

**Authors:** Namrata Sanjeevi, Luciana E. Hebert, Bidisha Mandal, Pablo Monsivais

**Affiliations:** ^1^ Department of Nutrition and Exercise Physiology, Elson S. Floyd College of Medicine Washington State University Spokane Washington USA; ^2^ Institute for Research and Education to Advance Community Health Washington State University Seattle Washington USA; ^3^ Elson S. Floyd College of Medicine Washington State University Spokane Washington USA; ^4^ School of Economic Sciences Washington State University Pullman Washington USA

**Keywords:** health care affordability, health care use, Medicaid, postpartum eligibility extension, unmet health care need

## Abstract

**Objectives:**

We examined the association of loss of pregnancy–related Medicaid coverage with unmet health care needs, medical financial hardship, and health care use in postpartum women participating in a nationally representative household survey.

**Design:**

Cross‐sectional study design.

**Sample and measurements:**

Using 2012–2018 National Health Interview Survey data, we classified postpartum women as either (1) having Medicaid coverage or (2) uninsured due to loss of pregnancy–related Medicaid coverage. We examined the relationship of loss of pregnancy–related Medicaid coverage with unmet health care needs, medical financial hardship, and health care use.

**Results:**

Compared to Medicaid‐insured postpartum women, uninsured women who lost pregnancy‐related Medicaid coverage had significantly greater odds of delaying medical care due to cost, as well as greater odds of unmet need for medical care, mental health care, and prescriptions. Uninsured postpartum women who lost pregnancy‐related Medicaid coverage also had significantly greater odds of medical financial hardship and lower odds of health care use.

**Conclusions:**

Findings suggest that continued Medicaid coverage during postpartum could improve health care access among uninsured women experiencing loss of pregnancy–related Medicaid eligibility. These results hold important implications for the public health impact of recent policy efforts to extend Medicaid eligibility into the postpartum period.

## Graphical abstract

In this study, uninsurance due to loss of pregnancy–related Medicaid coverage was significantly associated with greater odds of delaying medical care due to cost, unmet need for health care, and medical financial hardship. Sustaining pregnancy‐related Medicaid coverage could improve the health care access of uninsured postpartum women.

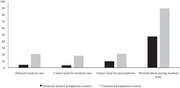

## 1 Background

Maternal mortality is higher in the United States compared to other developed nations [1], with an estimated rate of 22.3 deaths per 100,000 live births in 2022 [2]. Using data from Maternal Mortality Review Committees (MMRCs) in 38 states, about 26.9% of pregnancy‐related deaths were estimated to occur in late postpartum (i.e., between 43 days and 1 year after delivery) in 2020 [3]. A report aggregating data from nine state‐based MMRCs indicated that 58.3% of late maternal deaths are preventable [4], with improved access to care as a potential approach toward reducing maternal mortality [4]. Medicaid plays a crucial role in financing health care for low‐income pregnant women. In most states, pregnancy status broadens Medicaid eligibility by raising the income level at which eligibility is defined [5, 6]; as a result, many low‐income women gain Medicaid eligibility, which they would otherwise not qualify for, upon becoming pregnant (i.e., pregnancy‐related Medicaid). Although pregnant individuals are eligible for Medicaid if their income is up to 138% of federal poverty level [7], states may choose to expand pregnancy‐related Medicaid coverage beyond this threshold. Since 1986, women have maintained their pregnancy‐related Medicaid eligibility for 60 days following childbirth [8, 9]. At the end of the 60‐day postpartum period, the Congressional Budget Office estimates about 45% of women become uninsured [10]. Loss of insurance coverage during the late postpartum period may result in significant barriers to accessing postpartum care, which in turn could contribute to maternal mortality and poorer postpartum health.

Passed in response to the COVID‐19 Public Health Emergency, the Families First Coronavirus Response Act (FFCRA) prevented states from disenrolling Medicaid beneficiaries during the public health emergency, thus allowing for Medicaid eligibility extensions through the first year postpartum [11]. To date, one study has compared Medicaid claims, pre‐ and post‐FFCRA implementation, in postpartum women and found greater use of health care services post‐FFCRA implementation [12]. However, data for this study were limited to a single‐Medicaid health plan in Texas. Further, overlap of the post‐FFCRA timeframe with the COVID‐19 pandemic suggests that some changes in health care services may be explained by the pandemic. Additionally, the use of Medicaid claims data is expected to underestimate health services utilization using non‐Medicaid payment sources, which could have been the predominant source among pre‐FFCRA postpartum women who lost coverage. Perceptions of unmet health care needs, health care use, and affordability following loss of pregnancy–related Medicaid coverage could complement the existing literature by providing novel information on inequities in health care access resulting from gaps in Medicaid coverage [13]. The goal of this study is to examine the association of loss of pregnancy–related Medicaid coverage with unmet health care needs, medical financial hardship, and health care use in a sample of postpartum women participating in a nationally representative household survey, using data from the National Health Interview Survey (NHIS) for years 2012–2018.

## 2 Methods

### 2.1 Data Source and Study Population

The NHIS is a cross‐sectional, nationally representative household survey of the noninstitutionalized US civilian population that collects data on a broad range of health topics. The current study used NHIS data from 2012 to 2018. Of note, we used NHIS data only up to 2018 due to the availability of survey items that facilitated the identification of women who were uninsured due to the loss of pregnancy–related Medicaid coverage. Similar to previous studies using NHIS data [14, 15], we utilized pregnancy status in the past 12 months to identify postpartum women. The primary analytic sample was restricted to women aged 18‐49 years who were (1) pregnant in the past 12 months and (2) either covered by Medicaid or uninsured due to termination of Medicaid/medical plan after pregnancy. Comparisons between Medicaid‐insured women and those who lost Medicaid coverage could help understand the impact of loss of Medicaid coverage; thus, we did not include women who were covered only via private insurance. Further, women who were pregnant at the time of survey were excluded. The resulting sample consisted of 843 postpartum women.

### 2.2 Exposure Variable

#### 2.2.1 Medicaid Coverage Status

The NHIS interviews participants on health insurance status and the type of health insurance coverage, based on which participants are assigned to enrollment recodes representing their health insurance plan. More information on the assignment of enrollment recodes is provided in the NHIS person file [16] and the survey description document [17]. Postpartum women who were assigned to the “Medicaid” enrollment recode were classified as having “Medicaid coverage” (*n* = 704). Among those without a known comprehensive health insurance plan, additional questions were asked to determine the reason for not having insurance coverage. Postpartum women reporting “Medicaid/medical plan stopped after pregnancy” were classified as uninsured due to termination of pregnancy‐related Medicaid coverage (*n* = 139).

### 2.3 Outcome Variables

#### 2.3.1 Unmet Need for Health Care

An affirmative response to the question, “During the past 12 months, has medical care been delayed for you because of worry about cost? ”, identified women who delayed medical care due to cost, whereas a negative response identified women who did not delay medical care. Similarly, women were classified as having an unmet need for medical care, mental health care, and prescription medicines if they responded affirmatively to the questions on whether they needed each type of care, respectively, in the past 12 months, but did not get it because they could not afford it.

#### 2.3.2 Medical Financial Hardship

Women responding “very worried” or “somewhat worried” to the question, “If you get sick or have an accident, how worried are you that you will be able to pay your medical bills?”, were classified as “worried about paying medical bills,” whereas those responding “not at all worried” were classified as “not worried.” An affirmative response to the question, “In the past 12 months did you/anyone in the family have problems paying or were unable to pay any medical bills,” identified women in families having “problems paying medical bills,” whereas a negative response identified women in families without any problems.

#### 2.3.3 Health Care Utilization

Response to the question on whether the participant “had seen or talked to general doctor about their own health during the past 12 months” was used to assess health care utilization.

### 2.4 Covariates

Sociodemographic characteristics treated as covariates include age, race/ethnicity (non‐Hispanic White; non‐Hispanic Black; Hispanic; other), union status (married/living with partner; single [including widowed, divorced, separated, and never married]), region of residence (Northeast; Midwest; South; West), ratio of family income to poverty threshold, highest educational attainment (high school/less than high school; some college/associate degree; bachelor's degree or higher), and employment status (employed in the past year; unemployed in the past year). These covariates were chosen a priori based on previous research related to the study variables of interest [15, 18, 19, 20].

### 2.5 Statistical Analysis

Analyses were performed using SAS (version 9.4; SAS Institute, Inc., Cary, NC, USA) software. Descriptive statistics were used to summarize sociodemographic characteristics and outcome measures by postpartum Medicaid coverage status. Logistic regression analyses examined associations of loss of pregnancy–related Medicaid coverage with unmet need for health care, health care use, and medical financial hardship. Regression analyses were adjusted for sociodemographic covariates. Because some Medicaid‐insured women (*n* = 38) were assigned an additional enrollment recode corresponding to private insurance, sensitivity analyses were conducted by excluding these women from the analytic sample. All analyses accounted for the complex sampling design and incorporated sample weights.

## 3 Results

### 3.1 Demographic Characteristics and Outcome Measures

Table 1 indicates the demographic characteristics of Medicaid‐insured postpartum women and uninsured postpartum women who lost Medicaid after pregnancy. About 37% of Medicaid‐insured postpartum women were from the South, whereas the proportion of uninsured postpartum women from the South was about 63%. About 41% and 65% of Medicaid‐insured postpartum women were non‐Hispanic Black and had educational attainment of high school/less than high school, respectively, whereas about 28% and 73% of uninsured postpartum women were non‐Hispanic Black and had educational attainment of high school/less than high school, respectively. Table 2 indicates the outcome measures for the two groups of postpartum women. About 21% of uninsured postpartum women reported delaying medical care compared to 4% of Medicaid‐insured postpartum women. Similarly, a greater proportion of postpartum women who were uninsured due to loss of pregnancy–related Medicaid coverage had unmet needs for medical care, unmet needs for mental health care, unmet needs for prescriptions, and worried about paying medical bills.

**TABLE 1:** Demographic characteristics of Medicaid‐insured postpartum women and uninsured postpartum women who lost Medicaid after pregnancy: NHIS 2012–2018.

	Medicaid‐insured postpartum women	Postpartum women who were uninsured due to loss of pregnancy–related Medicaid coverage
Characteristics^a^	*n* = 704^b^	*n* = 139^b^
Age	26.3 ± 0.3	27.1 ± 0.6
Ratio of family income to poverty	1.29 ± 0.05	1.16 ± 0.07
Race/Ethnicity		
Non‐Hispanic White	188 (26.9)	85 (55.5)
Non‐Hispanic Black	264 (41.4)	30 (27.6)
Hispanic	194 (25.2)	20 (14.7)
Other	54 (6.5)	4 (2.2)
Union status		
Married/Living with partner	346 (56.5)	94 (69.1)
Widowed/Divorced/Separated/Never married	357 (43.5)	45 (30.9)
Highest educational attainment		
High school/less than high school	444 (65.2)	99 (72.7)
Some college/associate degree	218 (28.6)	38 (25.8)
Bachelor's degree or higher	41 (6.2)	2 (1.4)
Employment status in past year		
Employed	427 (59.1)	69 (51)
Unemployed	277 (40.9)	70 (49)
Region of residence		
Northeast	105 (16.2)	9 (4.9)
Midwest	150 (23.2)	16 (10.8)
South	260 (36.8)	81 (63.2)
West	189 (23.9)	33 (21.1)

Abbreviation: NHIS, National Health Interview Survey.

^a^ Data represented as mean ± standard error of mean or unweighted *n* (%).

^b^ Due to missingness, cell sizes for some demographic characteristics will not sum to the group totals.

**TABLE 2:** Unmet need for health care, medical financial hardship, and health care use of Medicaid‐insured postpartum women and uninsured postpartum women who lost Medicaid after pregnancy: NHIS 2012–2018.

	Medicaid‐insured postpartum women	Postpartum women who were uninsured due to loss of pregnancy–related Medicaid coverage
Outcome measures^a^	*n* = 704^b^	*n* = 139^b^
Delayed medical care		
Yes	30 (4.3)	30 (20.5)
No	674 (95.7)	109 (79.5)
Unmet need for medical care		
Yes	28 (3.5)	26 (18)
No	676 (96.5)	113 (82)
Unmet need for mental health care		
Yes	22 (3)	8 (6.3)
No	674 (97)	130 (93.7)
Unmet need for prescriptions		
Yes	70 (9.7)	28 (21)
No	626 (90.3)	110 (79)
Worried about paying medical bills		
Yes (very/somewhat)	342 (47)	123 (89.4)
No	354 (53)	15 (10.6)
From family having problems paying medical bills		
Yes	149 (20.5)	50 (37.3)
No	555 (79.5)	89 (62.7)
Seen/Talked to general doctor about health		
Yes	449 (66)	63 (41.5)
No	245 (34)	75 (58.5)

Abbreviation: NHIS, National Health Interview Survey.

^a^ Data represented as unweighted *n* (%).

^b^ Due to missingness, cell sizes for some outcome measures will not sum to the group totals.

### 3.2 Relationship of Medicaid Coverage Status With Unmet Need for Health Care

Table 3 indicates the association of Medicaid coverage status with unmet need for health care among postpartum women, adjusting for age, race/ethnicity, union status, region of residence, ratio of family income to poverty, highest educational attainment, and employment status. Compared to Medicaid‐insured postpartum women, uninsured women who lost pregnancy‐related Medicaid coverage had significantly greater odds of delaying medical care due to cost. In addition, uninsurance due to loss of pregnancy–related Medicaid coverage was significantly associated with greater odds of unmet need for medical care, mental health care, and prescriptions because of unaffordability. The associations did not change substantially when Medicaid‐insured women who were additionally enrolled in private insurance were excluded from the study sample.

**TABLE 3:** Association^a^ of loss of pregnancy–related Medicaid coverage with unmet need for health care in postpartum women (*n* = 843): NHIS 2012‐2018.

	Delayed medical care due to cost	Unmet need for medical care	Unmet need for mental health care	Unmet need for prescriptions
	OR (95% CI)	OR (95% CI)	OR (95% CI)	OR (95% CI)
Medicaid‐insured (reference category)	—	—	—	—
Uninsured due to loss of pregnancy–related Medicaid coverage	7.46 (3.30, 16.87)	7.01 (2.85, 17.12)	4.19 (1.56, 11.27)	2.16 (1.04, 4.46)

Abbreviations: CI, confidence interval; NHIS, National Health Interview Survey; OR, odds ratio.

^a^Overall regression models adjusted for age, race/ethnicity, union status, region of residence, ratio of family income to poverty, highest educational attainment, and employment status.

### 3.3 Relationship of Medicaid Coverage Status With Medical Financial Hardship and Health Care Use

Table 4 shows the association of Medicaid coverage status with medical hardships and health care use, adjusting for age, race/ethnicity, union status, region of residence, ratio of family income to poverty, highest educational attainment, and employment status. Compared to Medicaid‐insured postpartum women, uninsured postpartum women who lost pregnancy‐related Medicaid coverage had significantly greater odds of worrying about paying medical bills and were in families having problems paying medical bills. Loss of pregnancy–related Medicaid coverage also was significantly related to lower odds of health care use, as indicated by lower odds of seeing/talking to a general doctor. The associations did not change substantially when Medicaid‐insured women who were additionally enrolled in private insurance were excluded from the study sample.

**TABLE 4:** Association^a^ of loss of pregnancy–related Medicaid coverage with medical financial hardship and health care use in postpartum women (*n* = 843): NHIS 2012–2018.

	Worry about paying medical bills	From family having problems paying medical bills	Seen/talked with a general doctor
	OR (95% CI)	OR (95% CI)	OR (95% CI)
Medicaid‐insured (reference category)	—	—	—
Uninsured due to loss of pregnancy–related Medicaid coverage	7.26 (3.35, 15.72)	2.67 (1.55, 4.59)	0.39 (0.24, 0.64)

Abbreviations: CI, confidence interval; NHIS, National Health Interview Survey; OR, odds ratio.

^a^ Overall regression models adjusted for age, race/ethnicity, union status, region of residence, ratio of family income to poverty, highest educational attainment, and employment status.

## 4 Discussion

In this study using NHIS data, about 16% of postpartum women in the study sample were uninsured owing to loss of pregnancy–related Medicaid coverage. Among uninsured women, more than half were from the Southern United States. Our study analyses suggest that uninsured postpartum women who lost pregnancy‐related Medicaid coverage were more likely to experience unmet need for medical care, mental health care, and prescriptions due to unaffordability. Taken together with previous research indicating adverse impact of unmet health care needs on health outcomes [21, 22], our study suggests that loss of pregnancy–related Medicaid coverage, and subsequent uninsurance, could place women at high risk for health complications during the postpartum period. Uninsured postpartum women who lost Medicaid coverage also were more likely to delay medical care due to cost and experience medical financial hardship. These findings may be attributed to the role played by Medicaid in improving health care affordability among low‐income individuals, as demonstrated by the association of Affordable Care Act (ACA) Medicaid expansion with reductions in out‐of‐pocket spending and financial burden [23, 24, 25].

In the current study, we also observed that loss of pregnancy–related Medicaid coverage was associated with lower health care utilization, a finding that is somewhat complementary to a previous study [12], indicating increased use of health services within 1 year postpartum, using the Texas Medicaid claims data, following FFCRA implementation. This result is also comparable with other research suggesting lower health service utilization in late postpartum versus prenatal period [26] and difficulties obtaining care for postpartum health issues [27] among women with Medicaid‐paid deliveries.

Collectively, our study findings suggest better health care access in Medicaid‐insured postpartum women compared to uninsured women who lost coverage. This inference is consistent with previous research suggesting the beneficial effects of enhanced Medicaid coverage on health care access, as demonstrated by improved access [28] in states that expanded Medicaid under the ACA, relative to non‐expansion states.

The results from this study are timely in view of the ending of the continuous enrollment provision in March 2023. Beginning in April 2022, the 2021 American Rescue Plan Act allowed states to provide Medicaid coverage up to 1 year postpartum via a state plan amendment [29]. As of this writing, five states (Arkansas, Idaho, Iowa, and Wisconsin) have not implemented extensions to postpartum Medicaid eligibility [29]. Although extension to postpartum coverage is pending legislation in Iowa, Idaho and Nevada are planning to implement a 12‐month extension, and Wisconsin has proposed a limited coverage extension [29]. Closing the Medicaid coverage gap will be vital for meeting health care demands and improving health care affordability and use among postpartum women residing in these states.

Results of this study must be interpreted in view of the limitations. The cross‐sectional study design limits inferences on causality. Further, given that the study sample included women who were pregnant in the last 12 months, it is possible that the outcome measures were reflective of their health care access and use both during pregnancy and postpartum. However, because the two groups of postpartum women in this study are expected to be covered by Medicaid during pregnancy, we do not anticipate the study findings to be explained by their prenatal health care access and use. The study data included a period prior to full implementation of the ACA in 2014 [30] in addition to post‐ACA implementation, during which changes in states’ adoption of Medicaid expansion occurred. Because nine states implemented Medicaid expansion from 2019 to 2023, and the expansion broadens income eligibility for parents, our study sample may not be directly comparable to women who lost pregnancy‐related Medicaid coverage in more recent years. Because the NHIS question for the study period does not directly assess whether participants had a recent live birth, the study sample also could have included women whose pregnancies ended in miscarriage, stillbirth, or abortion. Further, those who lose Medicaid eligibility postpartum could have higher incomes compared to those with sustained coverage [31]. However, in our study sample, the ratio of family income to poverty was lower among women who lost pregnancy‐related Medicaid coverage. This finding could be attributed to the lack of inclusion of postpartum women who transitioned to private insurance. Further, although the study sample did not include women who transitioned to private insurance, the uninsurance rate in this study is lower than previous estimates of uninsurance following termination of pregnancy‐related Medicaid eligibility [10, 31]. Despite these limitations, the study has notable strengths. Specificity in NHIS question wording enabled identifying women who were uninsured due to loss of pregnancy–related Medicaid coverage. Adjustment of several hypothesized covariates in the regression analyses supports the internal validity of the results. In this study, race/ethnicity served as a proxy measure [32] for structural racism, which is a key contributor to racial/ethnic disparities in health care access [33, 34]. Future studies examining the study associations by racial group could provide an understanding of racial/ethnic disparities in the relationship between uninsurance due to loss of pregnancy–related Medicaid coverage and health care access. By assessing unmet health care needs, we provide a novel understanding of disparities in the required services and services actually received [35] among uninsured postpartum women experiencing gaps in Medicaid coverage.

### 4.1 Implications for Public Health

In this study, uninsured postpartum women who lost pregnancy‐related Medicaid coverage were more likely to experience unmet need for health care, medical financial hardship, and lower health care use than Medicaid‐insured women. The current study findings hold implications for public health. With access to health care considered as a social determinant of health that impacts health outcomes, the study findings imply unmet health care needs and worse health status among postpartum women residing in states that have not extended Medicaid coverage. Our analyses suggest that maintaining Medicaid coverage could improve postpartum health care access among women who would otherwise be uninsured due to loss of pregnancy–related Medicaid eligibility.

## Author Contributions

Namrata Sanjeevi conceptualized the study, conducted the analyses, and wrote the article. Bidisha Mandal, Luciana E. Hebert, and Pablo Monsivais provided feedback on variables to be used, interpreted the results, reviewed, edited, and re‐wrote the article.

## Ethics Statement

Due to the use of publicly available, de‐identified datasets in the current research, the study is considered as “not human subjects research” and did not require IRB approval.

## Consent

The authors used publicly available National Health Interview Survey (NHIS) data. The NHIS survey description document (https://nhis.ipums.org/nhis/resources/srvydesc2015.pdf) indicates that verbal consent for survey participation was obtained from respondents.

## Conflicts of Interest

The authors declare no conflicts of interest.

## Permission to Reproduce Material

Not applicable

## Trial Registration

Not applicable

## Data Availability

Data are publicly available at https://www.cdc.gov/nchs/nhis/data‐questionnaires‐documentation.htm.
